# Validation of Risk Models for Predicting Post‐SVR HCC in Real‐World Surveillance Across Global Geographic Regions

**DOI:** 10.1111/liv.70734

**Published:** 2026-06-08

**Authors:** Hidenori Toyoda, Yujin Hoshida, Neehar D. Parikh, Prasun K. Jalal, Federico Piñero, Manuel Mendizabal, Ezequiel Ridruejo, Hugo Cheinquer, Andrea Casadei‐Gardini, Mounika Kanneganti, Arndt Weinmann, Markus Peck‐Radosavljevic, Jean‐Francois Dufour, Pompilia Radu, Gamal Shiha, Riham Soliman, Shiv K. Sarin, Manoj Kumar, Jing‐Houng Wang, Pisit Tangkijvanich, Wattana Sukeepaisarnjaroen, Masanori Atsukawa, Haruki Uojima, Akito Nozaki, Makoto Nakamuta, Koichi Takaguchi, Atsushi Hiraoka, Hiroshi Abe, Kentaro Matsuura, Tsunamasa Watanabe, Noritomo Shimada, Kunihiko Tsuji, Toru Ishikawa, Shigeru Mikami, Ei Itobayashi, Philip J. Johnson, Amit G. Singal

**Affiliations:** ^1^ Department of Gastroenterology Ogaki Municipal Hospital Ogaki Japan; ^2^ Division of Digestive and Liver Diseases, Department of Internal Medicine University of Texas Southwestern Medical Center Dallas Texas USA; ^3^ Department of Internal Medicine University of Michigan Ann Arbor Michigan USA; ^4^ Division of Abdominal Transplantation, Department of Medicine Baylor College of Medicine Houston Texas USA; ^5^ Hospital Universitario Austral, School of Medicine Austral University Buenos Aires Argentina; ^6^ CEMIC, Centro de Educación Medica e Investigaciones Clinicas, Norberto Quirno Buenos Aires Argentina; ^7^ Gastroenterology and Hepatology Division Universidad de Federal Do Rio Grande Do Sul Porto Alegre Brazil; ^8^ Department of Medical Oncology Vita‐Salute San Raffaele University Milan Italy; ^9^ Department of Internal Medicine I University Medical Center of the Johannes Gutenberg University Mainz Germany; ^10^ Innere Medizin Und Gastroenterologie Klinikum Klagenfurt Am Worthersee Klagenfurt Austria; ^11^ Hepatology‐Department of Biomedical Research University of Bern Bern Switzerland; ^12^ Hepatology and Gastroenterology Unit, Internal Medicine Department, Faculty of Medicine Mansoura University and Egyptian Liver Research Institute and Hospital (ELRIAH) El Mansoura Egypt; ^13^ Tropical Medicine Department, Faculty of Medicine Port Said University Port Said Egypt; ^14^ Department of Hepatology Institute of Liver and Biliary Sciences New Delhi India; ^15^ Division of Hepatogastroenterology, Department of Internal Medicine Kaohsiung Chang Gung Memorial Hospital, Chang Gung University College of Medicine Kaohsiung Taiwan; ^16^ Center of Excellence in Hepatitis and Liver Cancer, Faculty of Medicine Chulalongkorn University Bangkok Thailand; ^17^ Division of Gastroenterology, Department of Medicine, Faculty of Medicine Khon Kaen University Khon Kaen Thailand; ^18^ Department of Internal Medicine, Division of Gastroenterology and Hepatology Nippon Medical School Tokyo Japan; ^19^ Department of Gastroenterology, Internal Medicine Kitasato University School of Medicine Sagamihara Japan; ^20^ Gastroenterology Center Yokohama City University Medical Center Yokohama Japan; ^21^ National Hospital Organization Kyushu Medical Center Fukuoka Japan; ^22^ Department of Hepatology Kagawa Prefectural Central Hospital Takamatsu Japan; ^23^ Gastroenterology Center Ehime Prefectural Central Hospital Matsuyama Japan; ^24^ Department of Internal Medicine, Division of Gastroenterology and Hepatology Shinmatusdo Central General Hospital Matsudo Japan; ^25^ Department of Gastroenterology and Metabolism Nagoya City University, Graduate School of Medical Sciences Nagoya Japan; ^26^ Department of Internal Medicine St. Marianna University School of Medicine Kawasaki Japan; ^27^ Department of Internal Medicine, Division of Gastroenterology and Hepatology Otakanomori Hospital Kashiwa Japan; ^28^ Center for Gastroenterology Teine Keijinkai Hospital Sapporo Japan; ^29^ Department of Hepatology Saiseikai Niigata Hospital Niigata Japan; ^30^ Department of Internal Medicine, Division of Gastroenterology Kikkoman General Hospital Noda Japan; ^31^ Department of Gastroenterology Asahi General Hospital Asahi Japan; ^32^ Department of Molecular and Clinical Cancer Medicine University of Liverpool Liverpool UK

**Keywords:** hepatitis C, hepatocellular carcinoma, real‐world, risk model, surveillance practice

## Abstract

**Background & Aims:**

Several clinical risk models have been proposed to stratify hepatocellular carcinoma (HCC) risk in patients with chronic hepatitis C virus (HCV) after sustained virologic response (SVR). However, validation efforts have focused on monocentric or country‐specific cohorts, and it is unclear if clinical risk models can be broadly applied to global populations. We characterised regional variation in model performance for HCC risk stratification in post‐SVR patients.

**Methods:**

Four HCC clinical risk models (aMAP score, FIB‐4 index, GES score, and Toronto HCC risk index [THRI]) were analysed in six real‐world cohorts, which included 8796 post‐SVR patients from different geographic regions globally. Model discrimination was assessed using Harrel's c‐statistic index. HCC incidence rates were compared across low‐, intermediate‐, and high‐risk groups for each model.

**Results:**

Distributions of patient characteristics and HCC incidence rates varied across geographic regions. Predictive performances of models were comparable within each cohort despite the model with the highest c‐statistics differing by regions. Performance was lower than those from original reports overall; c‐statistics of models across most regions remained below 0.70.

**Conclusions:**

There remains a continued need to improve discrimination and calibration of clinical models to stratify HCC risk in post‐SVR patients. Accuracy of models may differ by geographic region, underscoring the importance of external validation to assess transportability of models and suggesting no single model can be universally applied.

## Lay Summary

The risk of hepatocellular carcinoma (HCC) remains elevated in patients with hepatitis C virus (HCV) even after the eradication of HCV. Stratifying the risk of HCC development in patients with cured HCV is crucial. There have been several models for predicting HCC development based on routine clinical variables. However, the predictive performances of all four models were suboptimal in real‐world clinical settings with large variations across global geographic regions.

**Abbreviations**
AASLD: American Association for the Study of Liver Diseases
APASL: Asian Pacific Association for the Study of the Liver
DAA: direct‐acting antiviral
EASL: the European Association for the Study of the Liver
HCC: hepatocellular carcinoma
HCV: hepatitis C virus
IQR: interquartile range
SVR: sustained virologic response
THRI: Toronto HCC risk index

## 1 Introduction

The use of oral direct‐acting antiviral (DAA) regimens as anti‐hepatitis C virus (HCV) therapy has improved antiviral efficacy and tolerability. Consequently, the proportion of patients who have achieved sustained virologic response (SVR) has markedly increased [1]. Although SVR significantly decreases HCC risk [2], studies have reported persistent HCC risk exceeding 1% per year in patients with cirrhosis [3], underscoring the importance of continued HCC surveillance [4]. However, there is variation in HCC risk among patients with cirrhosis, as well as HCC developing in some patients without cirrhosis. In this context, assessing HCC risk after SVR is important for effective HCC surveillance implementation.

Several clinical risk models have been proposed to stratify HCC risk after SVR, with each showing promising performance when locally validated; however, it is unclear if performance would vary regionally and if these models would work in real‐world clinical practice of the respective region. In addition to differences in demographics and clinical characteristics between cohorts, there is a variation in post‐SVR surveillance practices [5, 6, 7], including candidate selection, surveillance intensity, and adherence. For example, criteria for identifying post‐SVR patients who warrant HCC surveillance differ across guidelines, particularly the required degree of liver fibrosis [5, 6, 7]. American Association for the Study of Liver Diseases (AASLD) guidelines and the European Association for the Study of the Liver (EASL) guidelines recommend post‐SVR HCC surveillance for only patients with cirrhosis [5, 6]. In contrast, Asian Pacific Association for the Study of the Liver (APASL) guidelines recommend surveillance for all SVR patients regardless of fibrosis [7]. Consequently, tumour burden and treatment receipt in post‐SVR patients who develop HCC widely vary by region/country [8]. These regional differences could influence the performance of risk stratification models, so it is unclear if one prediction model can be applied universally or if region‐specific models are needed. In addition to variation by region, the applicability of these models to real‐world practice for management of post‐SVR patients would also be unclear.

Herein, we leveraged an international collaboration combining several real‐world cohorts to evaluate and compare the discrimination of HCC risk stratification models for predicting HCC in patients with hepatitis C who achieved SVR.

## 2 Patients and Methods

### 2.1 Study Patients

We enrolled patients who were confirmed as having achieved SVR by DAA therapy (i.e., absence of serum HCV RNA at least 12 weeks after end of DAA therapy) from 30 high‐volume academic centers in Europe (Italy, Germany, Austria, and Switzerland), North America (USA), South America (Argentine and Brazil), Middle East (Egypt), South and Southeast Asia (India and Thailand), or East Asia (Japan and Taiwan). Baseline demographic and laboratory data prior to the start of DAA therapy and outcomes including the development of HCC were collected. In principle, patients were followed up after SVR according to the guidelines by academic societies of respective region [5, 6, 7, 9, 10, 11, 12]. Patients with a previous history of HCC, patients with presence of HCC or suspicious liver nodules on imaging prior to DAA initiation were excluded. Cirrhosis was determined by non‐invasive markers of fibrosis (e.g., transient elastography or Fibrosure), biopsy or imaging showing a cirrhotic‐appearing liver and signs of portal hypertension (e.g., gastroesophageal varices, collateral veins on imaging, or splenomegaly).

The study protocol was approved by the institutional review board of Ogaki Municipal Hospital and by the IRB of each participating institution, with a waiver of written informed consent. All procedures adhered to the Declaration of Helsinki.

### 2.2 Post‐SVR Surveillance and Diagnosis of HCC

Study patients were surveyed for HCC after achievement of SVR. In principle, the surveillance was according to the guidelines of the regions of the cohort. Recall policies for missed surveillance intervals were institutionally determined. The diagnosis of HCC was based on guidelines of academic societies for each participating country, typically consisting of histological confirmation or characteristic radiological imaging features (i.e., arterial phase hyperenhancement and delayed phase washout).

### 2.3 Calculations of HCC Risk Models and Categorisations of Risk Groups

We assessed four reported models for stratifying the risk of HCC: aMAP risk score [13], FIB‐4 index [14], GES score [15], and Toronto HCC risk index (THRI) [16]. All patients were categorised into three risk groups, low‐risk, intermediate‐risk, and high‐risk groups using the predefined threshold for each model (Table S1). These HCC risk stratification models were selected among the many reported HCC risk models [13, 14, 15, 16, 17, 18, 19, 20, 21, 22, 23, 24, 25] given their components are routinely available in clinical practice regardless of regions and the same numbers of risk categories (three categories) across models.

### 2.4 Statistical Analysis

Continuous variables are expressed as medians with interquartile ranges (IQRs) and categorical variables are expressed as numbers and percentages. The discriminative ability of each model was first assessed visually by plotting the Kaplan–Meier estimate of the incidence of HCC for each group, and the results were then compared with the log‐rank test. For HCC incidence, time zero was defined as the date of SVR‐12. Patients who did not develop HCC were censored at the last follow‐up. Discriminative ability was assessed using the concordance index, specifically the standard Harrell's c‐index, for which higher values indicate better discrimination. A c‐index of 0.50 indicates no discrimination, whereas a c‐index of 1.0 indicates perfect discrimination. In addition to discrimination, model calibration was assessed using Brier scores and calibration plots to evaluate agreement between predicted and observed HCC risk. Clinical utility was examined using decision curve analysis (DCA), estimating net benefit across a range of clinically relevant threshold probabilities compared with ‘treat‐all’ and ‘treat‐none’ strategies. These analyses were performed overall and by geographic regions.

Data analysis was performed using JMP statistical software, version 11.0.0 (Macintosh version; SAS Institute, Cary, NC) and EZR (Saitama Medical Center, Jichi Medical University, Saitama, Japan), the latter for which is a graphical user interface for R (The R Foundation for Statistical Computing, Vienna, Austria).

## 3 Results

### 3.1 Characteristics of Study Patients by Regions

Table 1 summarises the background characteristics of the study patients, stratified by region. There were regional differences in patient age, gender, HCV genotype, and proportions of patients with cirrhosis. The median age of patients at SVR was highest in East Asia (70 years) and lowest in South and Southeast Asia (52 years), between 55 and 60 years in other regions. Whereas male gender was predominant in Europe, North America, and South and Southeast Asian, female was predominant in South America and East Asia. The predominant HCV genotype was genotype 1 in Europe, North and South America, and East Asia, genotype 3 in South and Southeast Asia, and genotype 4 in the Middle East. The percentages of patients with cirrhosis in the cohort were between 50% and 65% in all regions except for South America (33.8%) and South and Southeast Asia (88.6%).

**TABLE 1:** Characteristics of patients with post‐SVR de novo HCC by regions.

	Total	Europe (Italy, Germany, Austria, Switzerland)	North America (USA)	South America (Argentine, Brazil)	Middle East (Egypt)	South and Southeast Asia (India, Thailand)	East Asia (Japan, Taiwan)
Number of patients	8796	838	1144	1990	1041	757	3026
Age (years)	62 (53–70)	59 (51–71)	61 (56–65)	60 (52–68)	56 (50–62)	52 (44–60)	70 (62–77)
Gender‐male	4425 (50.3)	487 (58.1)	671 (58.8)	939 (47.2)	524 (50.3)	510 (67.4)	1294 (42.8)
HCV genotype (1/2/3/4/5/6/mix^a^)	5428 (61.8)/1111 (12.7)/1003 (11.4)/1158 (13.2)/1 (0)/70 (0.8)/8 (0.1)	574 (68.6)/64 (7.7)/145 (17.3)/50 (6.0)/1 (0.1)/3 (0.4)/0	933 (81.7)/91 (8.0)/75 (6.6)/25 (2.2)/0/13 (1.1)/5 (0.4)	1433 (72.5)/203 (10.3)/308 (15.6)/33 (1.7)/0/0/0	0/0/0/1041 (100)/0/0/0	250 (33.0)/5 (0.7)/471 (62.2)/8 (1.1)/0/23 (3.0)/0	2238 (74.0)/748 (24.7)/4 (0.1)/1 (0.0)/1 (0.0)/31 (1.0)/3 (0.1)
Cirrhosis	4274 (54.4)	434 (56.7)	620 (56.4)	673 (33.8)	666 (64.0)	671 (88.6)	1660 (54.9)
Follow‐up period (months)	31.7 (21.0–41.7)	31.8 (22.0–39.1)	42.7 (24.0–59.6)	21.9 (12.9–32.5)	31.3 (26.6–34.9)	27.4 (16.4–36.6)	40.1 (27.5–57.5)
HCC development	541 (6.2)	50 (6.0)	34 (3.0)	42 (2.1)	35 (3.4)	92 (12.2)	288 (9.5)
Maximal HCC size (cm)	2.2 (1.5–3.8)	3.2 (2.2–5.1)	2.5 (1.6–3.7)	2.5 (2.0–4.3)	2.6 (2.0–5.0)	4.0 (2.0–8.0)	1.8 (1.3–2.5)

*Note:* Data are expressed as medians (interquartile range) or numbers (percentage).

^a^ Genotypes 1+2, 1+3, or 1+6.

### 3.2 HCC Incidence After SVR, Stratified by Region

Patients were followed up as medians of 21.9–42.7 months. Figure S1 demonstrates the cumulative incidence of HCC after SVR, stratified by region. The highest incidence of HCC was observed in South and Southeast Asia over the first 3 years; however, the cumulative incidences were similar at 5 years across cohorts, outside of lower incidence in the European cohort. We also observed variations in the maximum diameter of HCC at diagnosis across regions, from 1.8 cm in East Asia to 4.0 cm in South and Southeast Asia, and in the percentage of early‐stage HCC (BCLC stage 0 or A), from 95.8% in East Asia to 52.5% in Europe. The one‐, three‐, and five‐year incidence were 2.8%, 6.7%, and 9.3% in Europe, 2.3%, 10.0%, and 13.2% in North America, 0.9%, 2.8%, and 16.8% in South America, 0.3%, 3.8%, and 16.2% in the Middle East, 6.6%, 16.5%, and 16.5% in South and Southeast Asia, and 1.9%, 7.0%, and 12.1% in East Asia.

### 3.3 Performance of HCC Risk Stratification Models After SVR by Regions

Table 2 showed the distribution of patients for each HCC risk stratification model in each cohort, outside of FIB‐4 index in the South American cohort which could not be calculated due to lack of required data. There were large variations in patient distributions by each risk model. For example, aMAP categorised less than 20% of each cohort as low risk, whereas GES categorised over 50% of most cohorts as low risk, outside of North America (37.3%) and South and Southeast Asia (34.3%). Similarly, aMAP and FIB‐4 both categorised over one‐third of each cohort as high risk, whereas THRI classified less than 15% as high risk.

**TABLE 2:** Distributions of patients according to respective risk stratification model by regions.

	Total	Europe (Italy, Germany, Austria, Switzerland)	North America (USA)	South America (Argentine, Brazil)	Middle East (Egypt)	South and Southeast Asia (India, Thailand)	East Asia (Japan, Taiwan)
aMAP score							
Low	785 (10.6)	132 (19.4)	168 (17.1)	216 (17.7)	159 (16.5)	47 (6.8)	63 (2.2)
Intermediate	2444 (33.0)	234 (34.3)	421 (42.8)	480 (39.3)	440 (45.6)	181 (26.3)	688 (24.0)
High	4181 (56.4)	316 (46.3)	395 (40.1)	527 (43.1)	365 (37.9)	460 (66.9)	2118 (73.8)
FIB‐4 index^a^							
Low	382 (7.0)	61 (13.1)	32 (10.7)	—	180 (17.6)	41 (5.4)	68 (2.4)
Intermediate	1527 (28.1)	161 (34.6)	112 (37.3)	—	414 (40.4)	170 (22.6)	670 (23.2)
High	3527 (64.9)	243 (52.3)	156 (52.0)	—	431 (42.1)	543 (72.0)	2154 (74.5)
GES score							
Low	3258 (58.7)	246 (50.8)	98 (37.3)	601 (74.6)	190 (55.2)	247 (34.3)	1876 (63.9)
Intermediate	979 (17.6)	140 (28.9)	84 (31.9)	66 (8.2)	90 (26.2)	78 (10.8)	521 (17.8)
High	1315 (23.7)	98 (20.3)	81 (30.8)	139 (17.3)	64 (18.6)	395 (54.9)	538 (18.3)
THRI risk index							
Low	1532 (19.5)	221 (29.4)	261 (23.9)	362 (27.4)	310 (30.2)	152 (20.2)	226 (7.8)
Intermediate	5361 (68.4)	457 (60.8)	734 (67.2)	797 (60.2)	652 (63.6)	533 (70.7)	2188 (75.7)
High	947 (12.1)	74 (9.8)	98 (9.0)	164 (12.4)	64 (6.2)	69 (9.2)	478 (16.5)

*Note:* Data are expressed as numbers (percentage).

^a^ FIB‐4 index could not be calculated in the South American cohort due to the unavailability of AST and ALT.

When focusing on patients in whom all four risk scores were available (all three risk scores in the South American cohort), the distribution patterns of the respective region did not differ markedly, although there was a trend of increases in prevalences of patients categorised to higher risk groups (Table S2).

Table 3 showed the number and the incidence of HCC in low, intermediate, and high‐risk groups by respective risk model in each cohort. There were trends of increasing HCC incidences from low‐risk to high‐risk groups for each respective model, with some exceptions. Figures S2–S7 show the cumulative incidence of post‐SVR HCC in each cohort, stratified by clinical risk model categories. Whereas HCC incidence of the intermediate risk group for aMAP score was similar to that of the high‐risk group in the North American and South American cohorts (Figures S3 and S4), it was similar to the low‐risk group in the Middle East cohort and South and Southeast Asia cohort (Figures S5 and S6). HCC incidence was not associated with risk groups by GES score in the North American and South American cohorts (Figures S3 and S4). Notably, only one patient (South and Southeast Asia cohort) developed post‐SVR HCC among all patients of the low‐risk group of the aMAP model.

**TABLE 3:** Numbers and incidences of post‐SVR HCC according to regions and risk stratification models.

	Total	Europe (Italy, Germany, Austria, Switzerland)	North America (USA)	South America (Argentine, Brazil)	Middle East (Egypt)	South and Southeast Asia (India, Thailand)	East Asia (Japan, Taiwan)
Total HCC	541 (6.2)	50 (6.0)	34 (3.0)	42 (2.1)	35 (3.4)	92 (12.2)	288 (9.5)
aMAP score							
Low	1 (0.1)	0	0	0	0	1 (2.1)	0
Intermediate	69 (2.8)	7 (3.0)	11 (2.6)	9 (1.9)	3 (0.7)	10 (5.5)	29 (4.2)
High	415 (9.9)	31 (9.8)	23 (5.8)	22 (4.1)	27 (7.4)	69 (15.0)	243 (11.5)
FIB‐4 index^a^							
Low	4 (1.1)	1 (1.6)	1 (3.1)	—	0	1 (2.4)	1 (1.5)
Intermediate	68 (4.5)	4 (2.5)	12 (10.7)	—	9 (2.2)	6 (3.5)	37 (5.5)
High	393 (11.1)	25 (10.2)	21 (13.5)	—	26 (6.0)	84 (15.5)	237 (11.0)
GES score							
Low	177 (5.4)	14 (5.7)	11 (11.2)	9 (1.5)	4 (2.1)	12 (4.9)	127 (6.8)
Intermediate	92 (9.4)	9 (6.4)	14 (16.7)	3 (4.6)	2 (2.2)	5 (6.4)	59 (11.3)
High	201 (15.3)	15 (15.3)	8 (9.9)	6 (4.3)	9 (14.1)	68 (17.2)	95 (17.7)
THRI risk index							
Low	17 (1.1)	1 (0.5)	1 (0.4)	3 (0.8)	1 (0.3)	4 (2.6)	7 (3.1)
Intermediate	367 (6.9)	28 (6.1)	27 (3.7)	23 (2.9)	28 (4.3)	69 (13.0)	192 (8.8)
High	122 (12.9)	9 (12.2)	6 (6.1)	7 (4.3)	6 (9.4)	18 (26.1)	76 (15.9)

*Note:* Data are expressed as numbers (percentage).

^a^ FIB‐4 index could not be calculated in South American cohort due to unavailability of AST and ALT.

Table 4 describes the Harrel's c‐indices of each model across cohorts, which ranged between 0.5 and 0.8; however, discrimination was below 0.70 for most models across most regions. When comparing models within each respective region, all 4 models showed comparable c‐index values but the model with the highest c‐index, usually suggesting the best performance, differed by regions. The models with the highest c‐index for post‐SVR HCC were aMAP score in the European and Middle East cohorts, GES score in the South American, South and Southeast Asian, and East Asian cohorts, and THRI in the North American cohort. However, the differences between HCC risk models were not statistically significant, except for a few select comparisons: aMAP score vs. THRI risk index in Middle East cohort, and aMAP score vs. GES score, FIB‐4 index vs. GES score, and GES score vs. THRI risk index in East Asia cohort (Table S3).

**TABLE 4:** Harrel's C index of respective risk stratification model by regions.

	Europe (Italy, Germany, Austria, Switzerland)	North America (USA)	South America (Argentine, Brazil)	Middle East (Egypt)	South and Southeast Asia (India, Thailand)	East Asia (Japan, Taiwan)
aMAP score	0.687	0.536	0.626	0.776	0.611	0.549
95% CI	0.630–0.744	0.448–0.624	0.540–0.712	0.735–0.817	0.566–0.656	0.524–0.574
Number	625	281	1189	963	560	2830
HCC	32	28	25	29	71	251
FIB‐4 index^a^	0.639	0.580	—	0.720	0.626	0.549
95% CI	0.565–0.713	0.488–0.672	—	0.667–0.773	0.593–0.659	0.525–0.573
Number	431	279	—	1023	621	2852
HCC	27	28	—	33	79	254
GES score	0.578	0.523	0.649	0.756	0.630	0.636
95% CI	0.478–0.678	0.425–0.621	0.514–0.784	0.611–0.901	0.579–0.681	0.593–0.679
Number	452	244	746	342	594	2893
HCC	32	27	13	13	78	260
THRI risk index	0.647	0.582	0.566	0.698	0.628	0.571
95% CI	0.586–0.708	0.502–0.662	0.488–0.644	0.637–0.759	0.585–0.671	0.532–0.610
Number	687	281	1288	1024	621	2852
HCC	32	28	27	33	79	254

*Note:* The model with highest C index of respective region was underlined.

^a^ FIB‐4 index could not be calculated in South American cohort due to unavailability of AST and ALT.

### 3.4 Sensitivity Analyses

Because patients who are recommended for undergoing post‐SVR surveillance by most guidelines are those with cirrhosis, we conducted a sensitivity analysis focusing on patients who had cirrhosis at SVR. Although the models with the highest c‐index changed in some cohorts, they remained comparable within each cohort and ranged between 0.5 and 0.8 and below 0.70 for most models across most regions (Table S4). When excluding patients in whom post‐SVR HCC was detected and diagnosed at advanced stage (BCLC 2–4), for whom effective surveillance had actually failed, the results were similar, but c‐indices slightly increased in comparison to those in the entire study patients (Table S5).

### 3.5 Calibration and Overall Prediction Accuracy

Overall prediction accuracy varied across models and geographic regions as assessed by Brier scores, with lower values indicating better combined calibration and discrimination. The model with the lowest Brier score varied across regions (Table S6). Calibration plots showed generally acceptable agreement between predicted and observed HCC risk across models, although the degree of calibration varied by region and risk range (Figure S8).

### 3.6 Clinical Utility Assessed by Decision Curve Analysis

Decision curve analysis demonstrated that all four HCC risk stratification models provided greater net clinical benefit than ‘treat‐all’ or ‘treat‐none’ strategies across clinically relevant threshold probabilities. At lower threshold probabilities, corresponding to more aggressive surveillance strategies, net benefit was similar across models. At higher thresholds, the GES score showed the greatest net benefit in the overall cohort. In contrast, region‐specific analyses showed heterogeneity in clinical utility of the respective model (Figure S9).

## 4 Discussion

In this study, we validated 4 proposed HCC risk stratification models with routinely available components in real‐world practice from six different regions worldwide. The results showed large variations in the performances of these models and the difficulty of using HCC risk models in real clinical settings. The distribution of patients categorised as low, intermediate, or high risk by each model varied across geographic regions. Although the discrimination of the clinical risk scores was comparable in each respective cohort, the model with the highest c‐index varied by region, with no single model that universally demonstrated the best performance. Whereas the low‐risk group by aMAP score showed low HCC incidence regardless of cohorts, no model was able to consistently identify high‐risk patients across all cohorts. These data highlight that clinicians and researchers should carefully consider which model, if any, would be most appropriate for their individual cohort when assessing HCC risk after SVR.

These differences in model performance may partly be related to variation in the characteristics of post‐SVR patients by regions, including patient age, HCV genotype, and percentage of cirrhosis [26]. In association with the variation of the characteristics of SVR patients, HCC cumulative incidence after SVR varied, with higher incidence rates noted in South/Southeast Asia shortly after SVR. However, cumulative incidence rates became similar across cohorts with longer follow‐up. This may have been partly due to the higher percentage of patients with cirrhosis in the cohort but also due to the potential failure to detect minute HCC nodules prior to the commencement of DAA therapy.

Regional variations are also likely related to differences in surveillance practices for post‐SVR patients. Surveillance guidelines after SVR differ; AASLD guidelines recommending surveillance using semi‐annual ultrasound plus AFP and restricting only to those with cirrhosis at SVR [6], whereas EASL guidelines recommend post‐SVR surveillance using ultrasound alone for patients with cirrhosis at SVR [5], and APASL guidelines recommend performing surveillance for all SVR patients using ultrasound and multiple tumour markers (des‐gamma‐carboxy prothrombin and AFP‐L3 fraction in addition to AFP) [7]. Similarly, recall policies for those who missed visits or the threshold for considering diagnostic evaluation also likely differ by region. Thus, differences in surveillance intensity and adherence may partly explain variation in model performance and tumour stage at diagnosis. Indeed, in association with the differences in surveillance practice, tumour burden or post‐SVR HCC varies by regions [8]. However, there remained variations in c‐index of respective models between regions even focusing on patients with cirrhosis or excluding patients with post‐SVR advanced‐stage HCC at diagnosis, that is, HCC that were missed for early detection. This indicated that the regional variations in risk‐stratification model performances were not only due to the regional differences in surveillance practice.

Beyond geographic variation, it was noteworthy that model discrimination was suboptimal, with the c‐statistic below 0.70 for each model across most regions in real‐world settings. This reflects the inherent complexity of post‐SVR HCC risk prediction and the limitations of models based solely on routine clinical variables. Each predictive model had been developed on the basis of regional patient populations. For example, the GES score has been developed based on Egyptian patients with SVR, whose HCV genotype was uniquely 4, which may limit the transportability of respective models across diverse populations. In addition, the FIB‐4 index originally reflects liver fibrosis, although liver fibrosis is one of the strongest factors associated with the development of HCC. Similarly, calibration showed no model was perfectly able to identify high and low‐risk patients. These data indicated the difficulty of applying the reported models in real‐world practice with the same performances as those in the original reports. All cohorts were based on real‐world clinical settings, and all patients may not have always been surveyed strictly according to the surveillance guidelines of the respective region or adhered to surveillance recommendations. Therefore, the suboptimal risk stratification performance with regional variations of the respective model was not always due to differences in the surveillance guidelines of post‐SVR patients of regions directly. The results highlight the need for improved risk stratification prior to implementation in clinical practice. The performance of included models may be low given methodological issues in model derivation [27], as well as each model only included readily available clinical variables, and more nuanced variables such as liver stiffness or blood‐based biomarkers may be of assistance [28, 29].

The extended performance analyses highlighted important regional differences in calibration, clinical utility, and reclassification performance of post‐SVR HCC risk models. Decision curve analysis demonstrated that all models provided meaningful clinical benefit compared with uniform surveillance strategies; however, the magnitude of benefit of using models differed by region. These findings further suggested that while risk‐stratified surveillance can improve efficiency in many regions, a uniform approach to post‐SVR risk prediction is unlikely to be optimal globally, and region‐adapted or population‐specific models may be required.

It is noteworthy that only one patient developed post‐SVR HCC in the low‐risk group of aMAP score; no patients developed HCC in all cohorts except for the cohort of South/Southeast Asia. This model may delineate a subgroup with negligible HCC risk and may help identify patients at very low risk, warranting further prospective evaluation for potential surveillance optimisation.

There were several limitations to this study. We validated only 4 risk‐stratification models for predicting post‐SVR HCC because the components for calculating scores of these models were feasible with routinely available laboratory tests and because the number of risk categories was the same. Other variables such as liver stiffness by transient elastography, the data of which were not available from all cohorts, may have improved model performance and could have been useful universally across regions. Second, the number of patients in the cohorts varied largely, from 757 patients in the South/Southeast Asian cohort to 3026 patients in the East Asian cohort, which may have influenced the performances of risk stratification models. Third, the definition of cirrhosis was not consistent across cohorts. Fourth, the competing risk of death during the study period was not considered in the analysis of HCC incidence due to the lack of information about death. Finally, there may have been a small variation in the way of data collection by cohorts due to the retrospective nature of data collection despite the fact that all cohorts were being followed up prospectively. However, all participating institutions of this study were high‐volume academic centers, and the influence of the variations in surveillance practice or data collection was minimal.

In conclusion, our results underscore the difficulty of applying risk stratification models for surveillance of post‐SVR HCC in real‐world clinical practice. The performance of risk stratification models to predict HCC development was suboptimal, and the ‘optimal model’ differed by region. Future studies should focus on improving the diagnostic performance of risk stratification models, as well as generalisability to broad international populations.

## Author Contributions

Concept and study design: Hidenori Toyoda and Amit G. Singal. Data acquisition: All authors. Data analysis: Hidenori Toyoda, Amit G. Singal., and Philip J. Johnson. Preparation of the manuscript: Hidenori Toyoda and Amit G. Singal. Data interpretation, review and/or revision of the manuscript: All authors.

## Funding

The authors have nothing to report. Dr. Singal's research is supported by NIH U01 CA283935, P50 CA295495, and U01 CA271887.

## Ethics Statement

The study protocol was approved by the institutional review board of Ogaki Municipal Hospital and by IRB of each participating institution, with waiver of written informed consent. All procedures adhered to the Declaration of Helsinki.

## Conflicts of Interest

The authors declare no conflicts of interest.

## Supporting information


**Figure S1:** Incidences of hepatocellular carcinoma (HCC) in patients with hepatitis C virus infection who achieved sustained virologic response (SVR) and with no history of HCC after SVR.
**Figure S2:** Incidences of hepatocellular carcinoma (HCC) in patients with hepatitis C virus infection who achieved sustained virologic response (SVR) and with no history of HCC after SVR based on serological models in European cohort. (A) By aMAP score, (B) By FIB‐4 index, (C) by GES score, and (D) THRI risk index. Green line, low‐risk group; blue line, intermediate‐risk group; red line, high‐risk group.
**Figure S3:** Incidences of hepatocellular carcinoma (HCC) in patients with hepatitis C virus infection who achieved sustained virologic response (SVR) and with no history of HCC after SVR based on serological models in North American cohort. (A) By aMAP score, (B) By FIB‐4 index, (C) by GES score, and (D) THRI risk index. Green line, low‐risk group; blue line, intermediate‐risk group; red line, high‐risk group.
**Figure S4:** Incidences of hepatocellular carcinoma (HCC) in patients with hepatitis C virus infection who achieved sustained virologic response (SVR) and with no history of HCC after SVR based on serological models in South American cohort. (A) By aMAP score, (C) by GES score, and (D) THRI risk index. FIB‐4 index could not be calculated in South American cohort due to unavailability of AST and ALT. Green line, low‐risk group; blue line, intermediate‐risk group; red line, high‐risk group.
**Figure S5:** Incidences of hepatocellular carcinoma (HCC) in patients with hepatitis C virus infection who achieved sustained virologic response (SVR) and with no history of HCC after SVR based on serological models in Middle East cohort. (A) By aMAP score, (B) By FIB‐4 index, (C) by GES score, and (D) THRI risk index. Green line, low‐risk group; blue line, intermediate‐risk group; red line, high‐risk group.
**Figure S6:** Incidences of hepatocellular carcinoma (HCC) in patients with hepatitis C virus infection who achieved sustained virologic response (SVR) and with no history of HCC after SVR based on serological models in South and Southeast Asian cohort. (A) By aMAP score, (B) By FIB‐4 index, (C) by GES score, and (D) THRI risk index. Green line, low‐risk group; blue line, intermediate‐risk group; red line, high‐risk group.
**Figure S7:** Incidences of hepatocellular carcinoma (HCC) in patients with hepatitis C virus infection who achieved sustained virologic response (SVR) and with no history of HCC after SVR based on serological models in East Asian cohort. (A) By aMAP score, (B) By FIB‐4 index, (C) by GES score, and (D) THRI risk index. Green line, low‐risk group; blue line, intermediate‐risk group; red line, high‐risk group.
**Figure S8:** Calibration plots comparing predicted and observed risk of hepatocellular carcinoma (HCC) after sustained virologic response (SVR) for aMAP score, FIB‐4 index, GES score, and THRI risk index. Predicted HCC risk is shown on the x‐axis and observed incidence on the y‐axis. The diagonal line represents perfect calibration. Overall, models demonstrated reasonable calibration, with variability across geographic regions and risk ranges.
**Figure S9:** Decision curve analysis (DCA) assessing the clinical utility of aMAP score, FIB‐4 index, GES score, and THRI risk index for prediction of hepatocellular carcinoma (HCC) after sustained virologic response (SVR). Net benefit is plotted across threshold probabilities and compared with ‘treat‐all’ and ‘treat‐none’ strategies. All models demonstrated greater net benefit than uniform strategies across clinically relevant thresholds, with the magnitude of benefit varying by models and regions. EA, East Asia; EU, Europe; LA, South America; ME, Middle East; NA, North America; SA‐SEA, South and Southeast Asia; amap, aMAP score; fib4, FIB‐4 index; ges, GES score; thri, THRI risk index.

**Table S1:** Factors for calculation and cut‐off of categorisation of risk groups by respective HCC risk model.
**Table S2:**. Distributions of patients according to respective risk stratification model by regions in patients in whom all four risk scores* were available.
**Table S3:** *p* values for comparison of C index between hepatocellular carcinoma risk models in each cohort.
**Table S4:** Harrel's C index of respective risk stratification model by regions focusing on patients with cirrhosis at SVR.
**Table S5:** Harrel's C index of respective risk stratification model by regions excluding patients with post‐SVR HCC detected and diagnosed at advanced stage (BCLC 2–4).
**Table S6:** Brier score of respective risk stratification model by regions.

## Data Availability

The data that support the findings of this study are available on request from the corresponding author. The data are not publicly available due to privacy or ethical restrictions.
