# Supplementary material for: Validation of Risk Models for Predicting Post‐SVR HCC in Real‐World Surveillance Across Global Geographic Regions
Source: Liver Int. 2026 Jun 8;46(7):e70734. doi: 10.1111/liv.70734 (PMC13244809; doi:10.1111/liv.70734)
Supplement: Supplementary file 1 — Figure S1: Incidences of hepatocellular carcinoma (HCC) in patients with hepatitis C virus infection who achieved sustained virologic response (SVR) and with no history of HCC after SVR. Figure S2: Incidences of hepatocellular carcinoma (HCC) in patients with hepatitis C virus infection who achieved sustained virologic response (SVR) and with no history of HCC after SVR based on serological models in European cohort. (A) By aMAP score, (B) By FIB‐4 index, (C) by GES score, and (D) THRI risk index. Green line, low‐risk group; blue line, intermediate‐risk group; red line, high‐risk group. Figure S3: Incidences of hepatocellular carcinoma (HCC) in patients with hepatitis C virus infection who achieved sustained virologic response (SVR) and with no history of HCC after SVR based on serological models in North American cohort. (A) By aMAP score, (B) By FIB‐4 index, (C) by GES score, and (D) THRI risk index. Green line, low‐risk group; blue line, intermediate‐risk group; red line, high‐risk group. Figure S4: Incidences of hepatocellular carcinoma (HCC) in patients with hepatitis C virus infection who achieved sustained virologic response (SVR) and with no history of HCC after SVR based on serological models in South American cohort. (A) By aMAP score, (C) by GES score, and (D) THRI risk index. FIB‐4 index could not be calculated in South American cohort due to unavailability of AST and ALT. Green line, low‐risk group; blue line, intermediate‐risk group; red line, high‐risk group. Figure S5: Incidences of hepatocellular carcinoma (HCC) in patients with hepatitis C virus infection who achieved sustained virologic response (SVR) and with no history of HCC after SVR based on serological models in Middle East cohort. (A) By aMAP score, (B) By FIB‐4 index, (C) by GES score, and (D) THRI risk index. Green line, low‐risk group; blue line, intermediate‐risk group; red line, high‐risk group. Figure S6: Incidences of hepatocellular carcinoma (HCC) in patients with hepatitis C virus [file LIV-46-0-s001.pptx]

## Slide 1
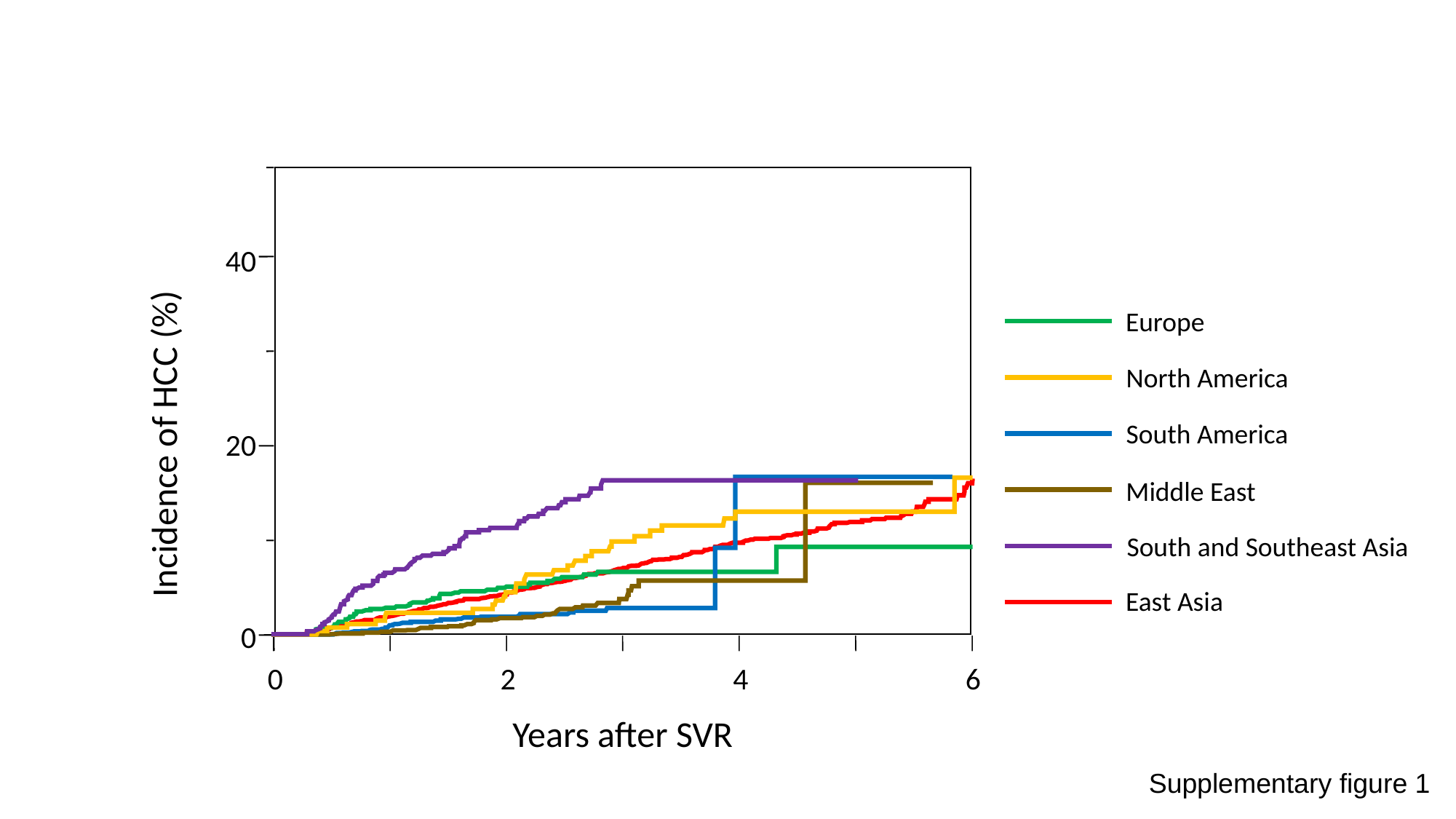

40
Europe
North America
South America
Incidence of HCC (%)
20
Middle East
South and Southeast Asia
East Asia
0
0
2
4
6
Years after SVR
Supplementary figure 1

## Slide 2
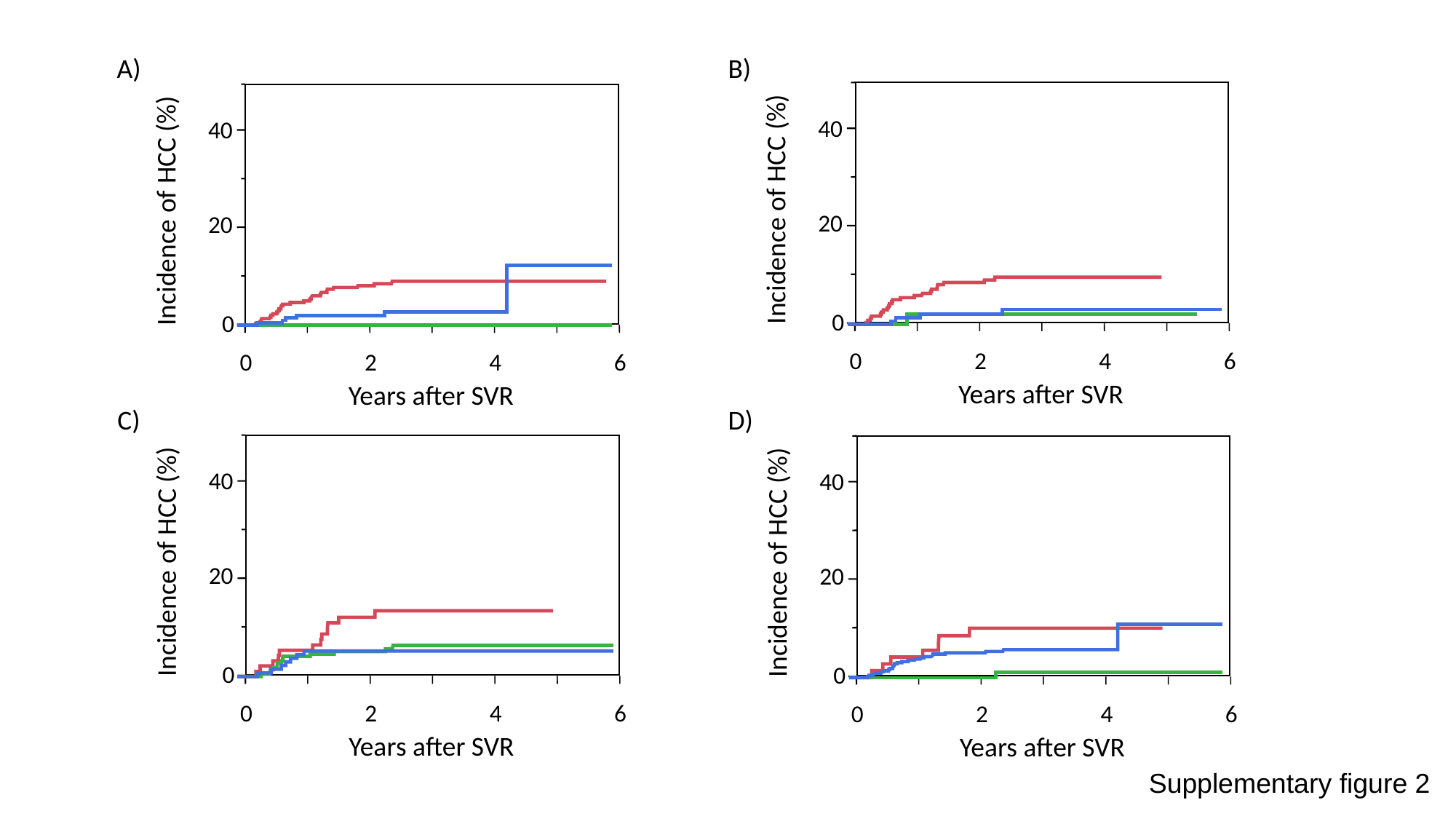

A)
B)
40
20
0
Incidence of HCC (%)
0
2
4
6
Years after SVR
40
20
0
Incidence of HCC (%)
0
2
4
6
Years after SVR
C)
D)
40
20
0
Incidence of HCC (%)
0
2
4
6
Years after SVR
40
20
0
Incidence of HCC (%)
0
2
4
6
Years after SVR
Supplementary figure 2

## Slide 3
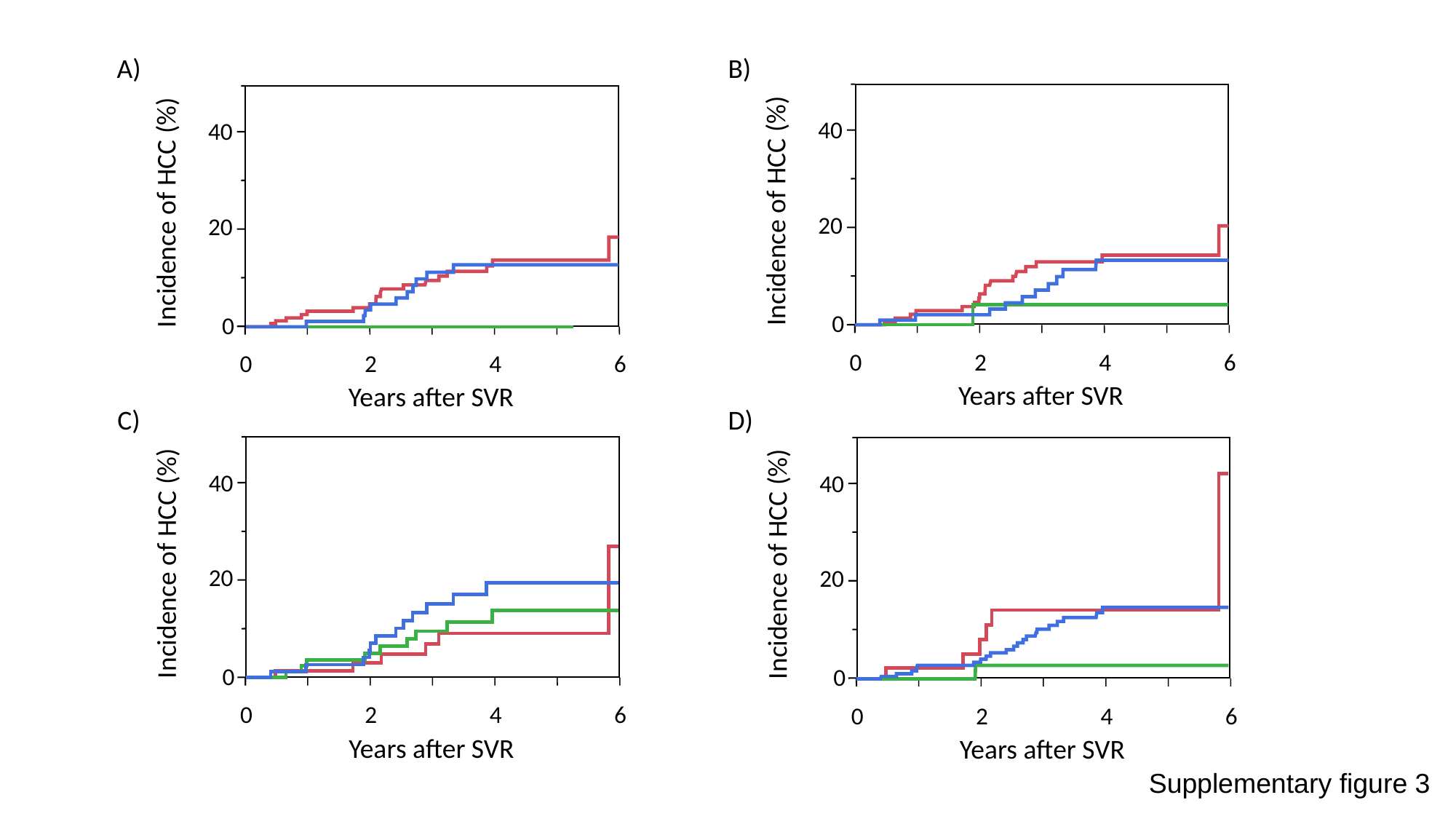

A)
B)
40
20
0
Incidence of HCC (%)
0
2
4
6
Years after SVR
40
20
0
Incidence of HCC (%)
0
2
4
6
Years after SVR
C)
D)
40
20
0
Incidence of HCC (%)
0
2
4
6
Years after SVR
40
20
0
Incidence of HCC (%)
0
2
4
6
Years after SVR
Supplementary figure 3

## Slide 4
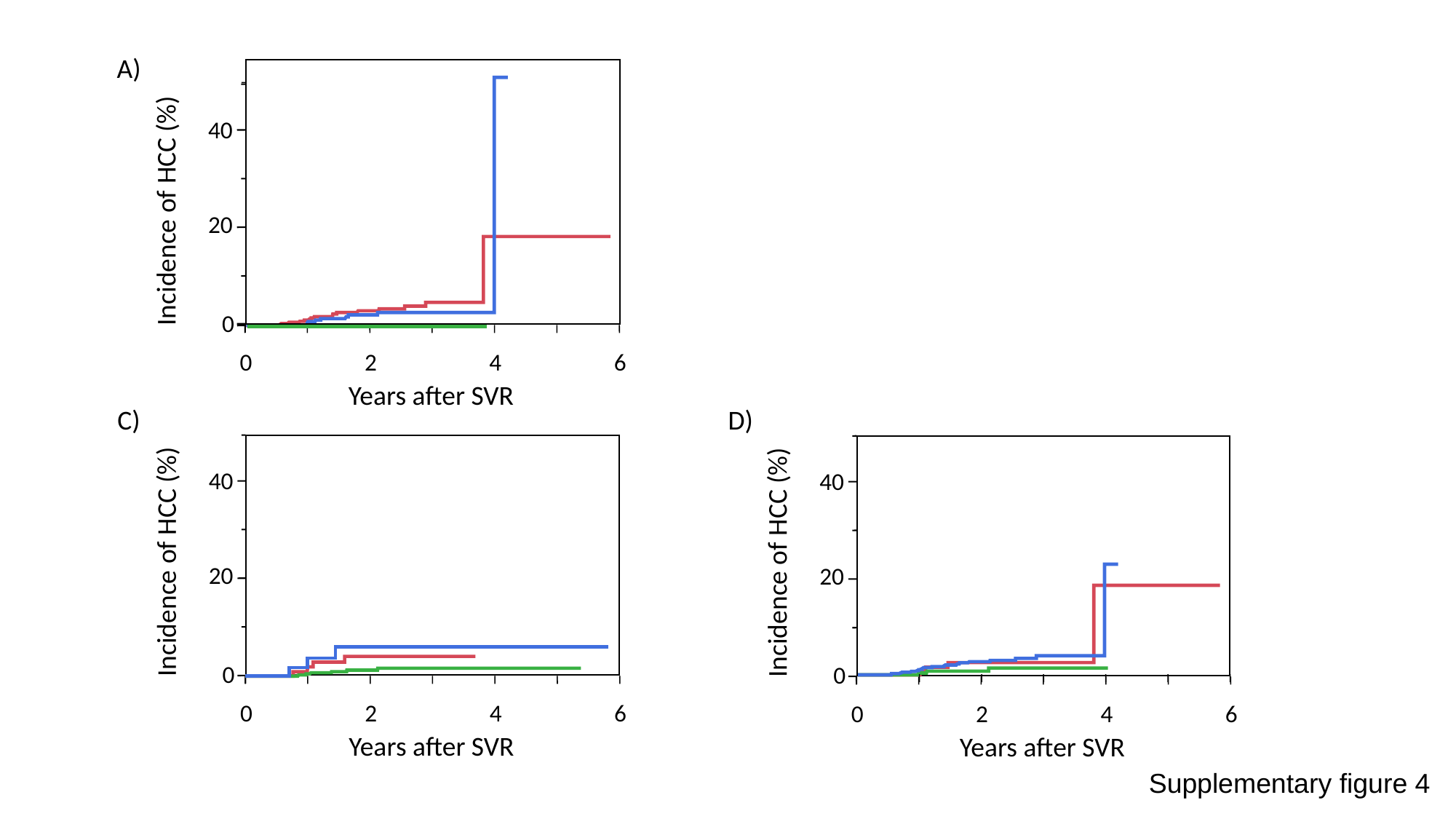

A)
40
20
0
Incidence of HCC (%)
0
2
4
6
Years after SVR
C)
D)
40
20
0
Incidence of HCC (%)
0
2
4
6
Years after SVR
40
20
0
Incidence of HCC (%)
0
2
4
6
Years after SVR
Supplementary figure 4

## Slide 5
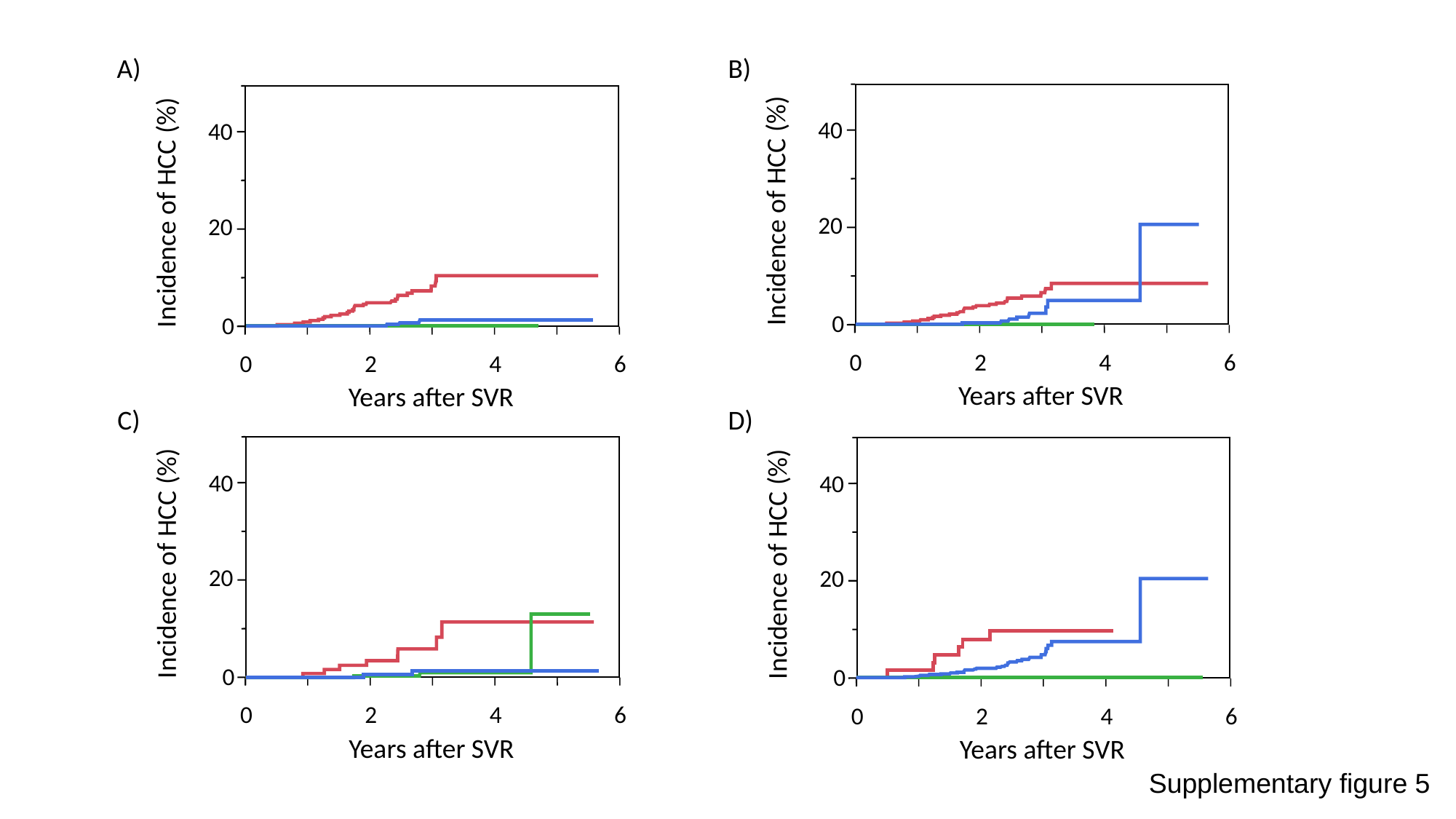

A)
B)
40
20
0
Incidence of HCC (%)
0
2
4
6
Years after SVR
40
20
0
Incidence of HCC (%)
0
2
4
6
Years after SVR
C)
D)
40
20
0
Incidence of HCC (%)
0
2
4
6
Years after SVR
40
20
0
Incidence of HCC (%)
0
2
4
6
Years after SVR
Supplementary figure 5

## Slide 6
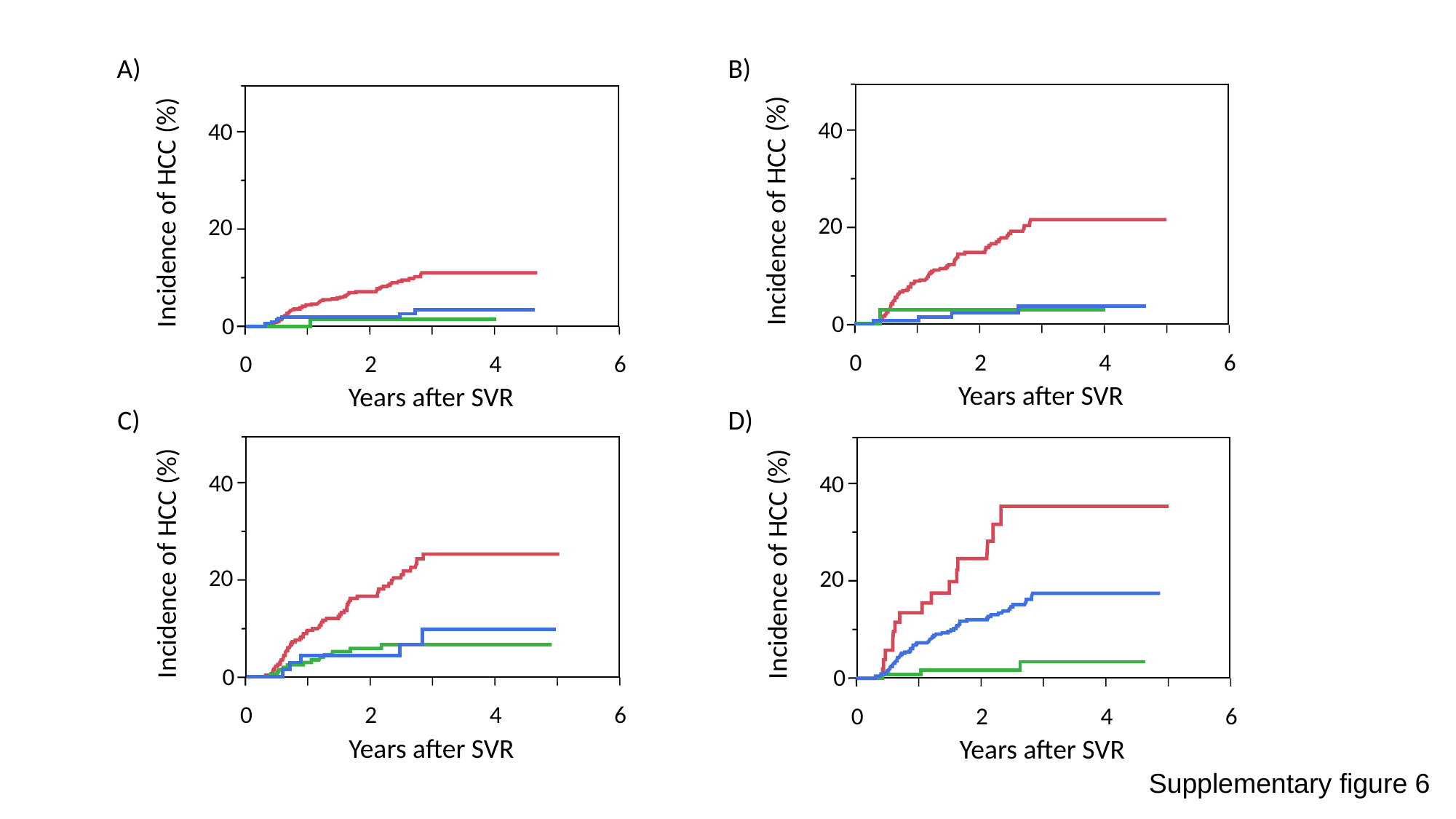

A)
B)
40
20
0
Incidence of HCC (%)
0
2
4
6
Years after SVR
40
20
0
Incidence of HCC (%)
0
2
4
6
Years after SVR
C)
D)
40
20
0
Incidence of HCC (%)
0
2
4
6
Years after SVR
40
20
0
Incidence of HCC (%)
0
2
4
6
Years after SVR
Supplementary figure 6

## Slide 7
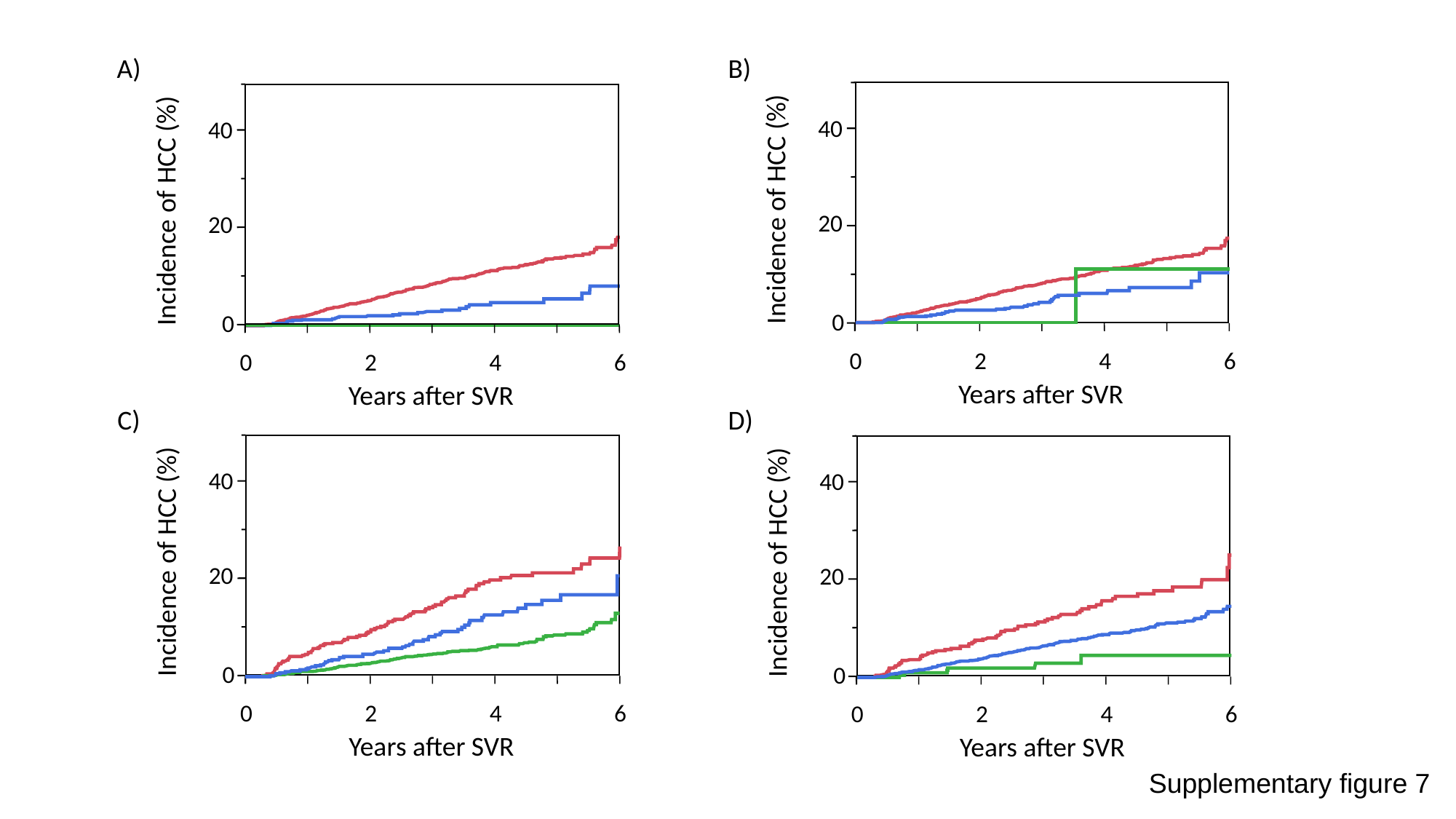

A)
B)
40
20
0
Incidence of HCC (%)
0
2
4
6
Years after SVR
40
20
0
Incidence of HCC (%)
0
2
4
6
Years after SVR
C)
D)
40
20
0
Incidence of HCC (%)
0
2
4
6
Years after SVR
40
20
0
Incidence of HCC (%)
0
2
4
6
Years after SVR
Supplementary figure 7

## Slide 8
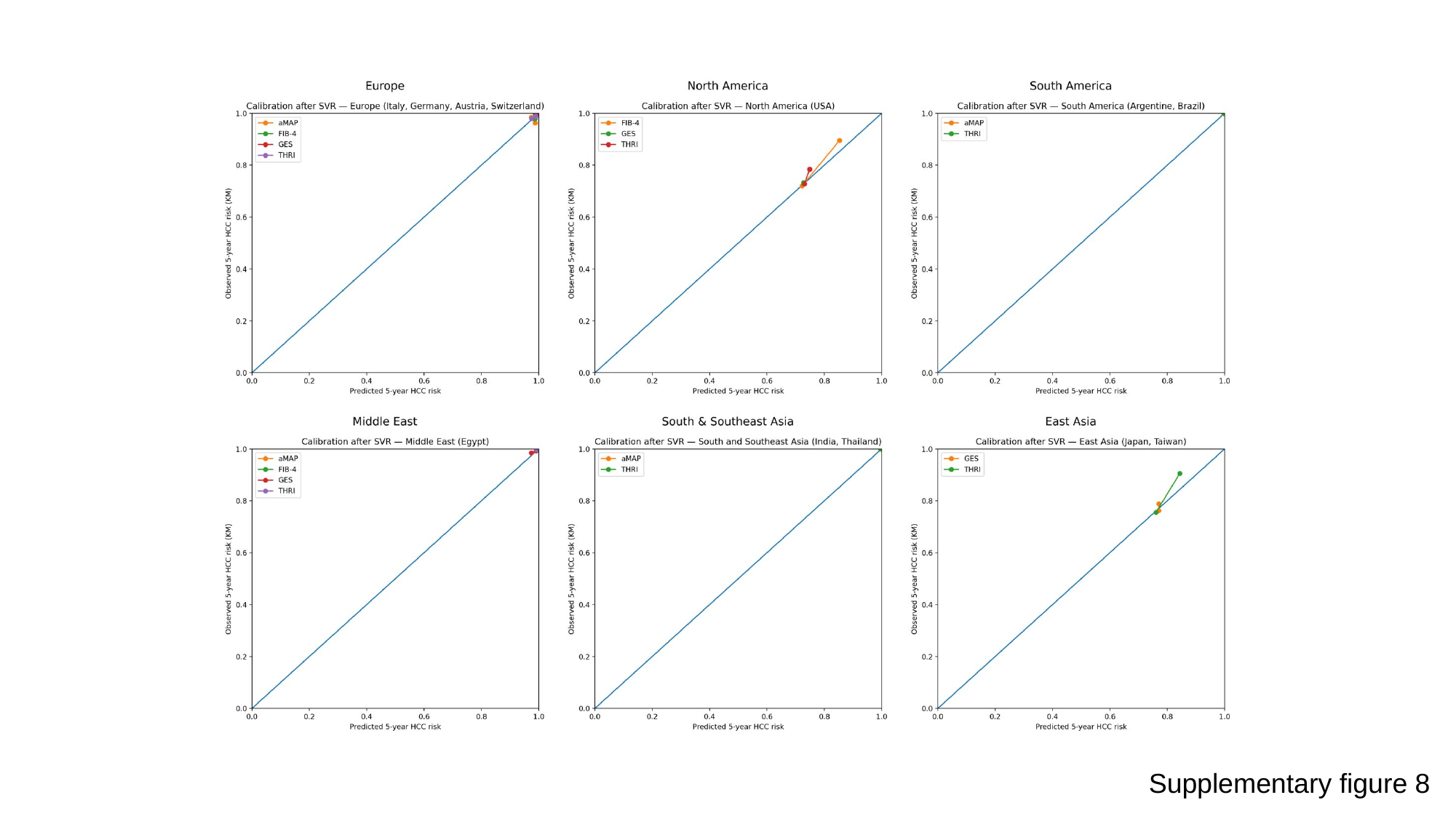

Supplementary figure 8

## Slide 9
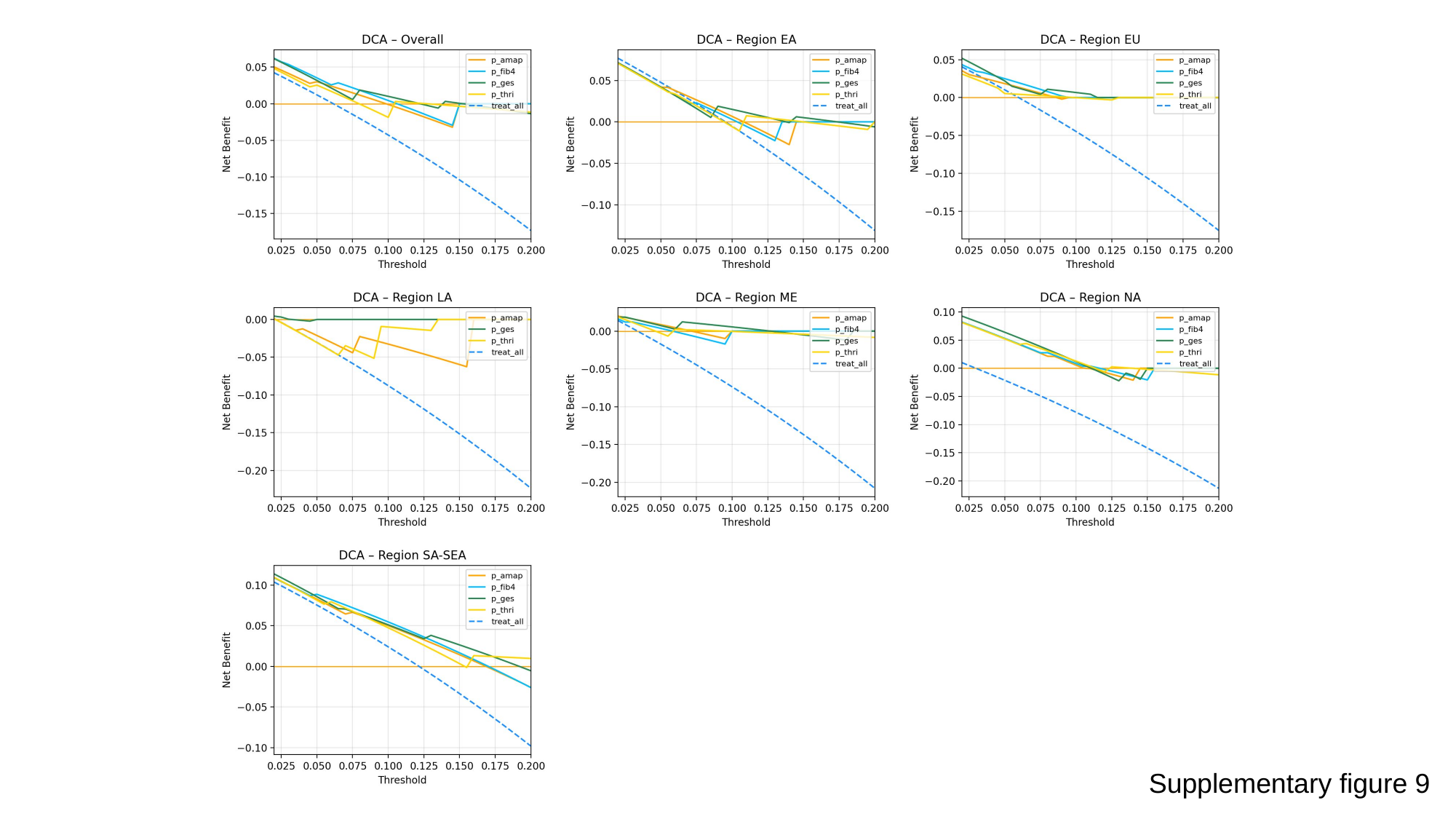

Supplementary figure 9
