# Supplementary material for: Validation of Risk Models for Predicting Post‐SVR HCC in Real‐World Surveillance Across Global Geographic Regions
Source: Liver Int. 2026 Jun 8;46(7):e70734. doi: 10.1111/liv.70734 (PMC13244809; doi:10.1111/liv.70734)
Supplement: Supplementary file 2 — Table S1: Factors for calculation and cut‐off of categorisation of risk groups by respective HCC risk model. Table S2:. Distributions of patients according to respective risk stratification model by regions in patients in whom all four risk scores* were available. Table S3: p values for comparison of C index between hepatocellular carcinoma risk models in each cohort. Table S4: Harrel's C index of respective risk stratification model by regions focusing on patients with cirrhosis at SVR. Table S5: Harrel's C index of respective risk stratification model by regions excluding patients with post‐SVR HCC detected and diagnosed at advanced stage (BCLC 2–4). Table S6: Brier score of respective risk stratification model by regions. [file LIV-46-0-s002.docx]

**Supplementary table 1**. Factors for calculation and cut-off of categorization of risk groups by respective HCC risk model.

| Models | Factors for calculation | Risk categorization | | |
| --- | --- | --- | --- | --- |
|  |  | Low risk | Intermediate risk | High risk |
| aMAP score | Age, gender, platelet counts, ALB, T-Bil | 0–50 | 50–60 | 60–100 |
| FIB-4 index | Age, platelet counts, AST, ALT | <1.45 | 1.45–3.25 | >3.25 |
| GES score | Age, gender, fibrosis, ALB, AFP | 0–6 | 6.5–7.5 | 8–12.5 |
| Toronto HCC risk index | Age, gender, etiology, platelet counts | <120 | 120–240 | >240 |

**Supplementary table 2**. Distributions of patients according to respective risk stratification model by regions in patients in whom all four risk scores* were available

|  | Total | Europe  (Italy, Germany, Austria, Switzerland) | North America  (USA) | South America  (Argentine, Brazil) | Middle East  (Egypt) | South and Southeast Asia  (India, Thailand) | East Asia  (Japan, Taiwan) |
| --- | --- | --- | --- | --- | --- | --- | --- |
| aMAP score |  |  |  |  |  |  |  |
| low | 304 (6.1) | 39 (9.8) | 11 (4.2) | 104 (18.3) | 42 (14.2) | 46 (6.9) | 62 (2.2) |
| intermediate | 1398 (27.9) | 123 (30.8) | 94 (35.7) | 198 (34.9) | 139 (47.0) | 172 (25.7) | 672 (23.9) |
| high | 3307 (66.0) | 237 (59.4) | 158 (60.1) | 266 (46.8) | 115 (38.9) | 451 (67.4) | 2080 (73.9) |
| FIB-4 index** |  |  |  |  |  |  |  |
| low | 219 (4.9) | 49 (12.3) | 22 (8.4) | --- | 46 (15.5) | 36 (5.4) | 66 (2.4) |
| intermediate | 1152 (25.9) | 135 (33.8) | 97 (36.9) | --- | 118 (39.9) | 150 (22.4) | 652 (23.2) |
| high | 3070 (69.1) | 215 (53.9) | 144 (54.8) | --- | 132 (44.6) | 483 (72.2) | 2096 (74.5) |
| GES score |  |  |  |  |  |  |  |
| low | 2918 (58.3) | 202 (50.6) | 98 (37.3) | 435 (76.6) | 149 (50.3) | 233 (34.8) | 1801 (64.0) |
| intermediate | 903 (18.0) | 119 (29.8) | 84 (31.9) | 52 (9.2) | 87 (29.4) | 58 (8.7) | 503 (17.9) |
| high | 1188 (23.7) | 78 (19.6) | 81 (30.8) | 81 (14.3) | 60 (20.3) | 378 (56.5) | 510 (18.1) |
| THRI risk index |  |  |  |  |  |  |  |
| low | 695 (13.9) | 76 (19.1) | 35 (13.3) | 141 (24.8) | 79 (26.7) | 144 (21.5) | 220 (7.8) |
| intermediate | 3586 (71.6) | 268 (67.2) | 186 (70.7) | 345 (60.7) | 195 (65.9) | 466 (69.7) | 2126 (75.6) |
| high | 728 (14.5) | 55 (13.8) | 42 (16.0) | 82 (14.4) | 22 (7.4) | 59 (8.8) | 468 (16.6) |

Data are expressed as numbers (percentage).

*All three risk scores except for FIB-4 index in South American cohort.

**FIB-4 index could not be calculated in South American cohort due to unavailability of AST and ALT.

**Supplementary table 3**. P values for comparison of C index between hepatocellular carcinoma risk models in each cohort.

|  | Europe | North America | South America | Middle East | South and Southeast Asia | East Asia |
| --- | --- | --- | --- | --- | --- | --- |
| aMAP score vs. FIB-4 index | 0.3156 | 0.4992 | --- | 0.1018 | 0.6001 | 1.0000 |
| aMAP score vs. GES score | 0.0636 | 0.8468 | 0.7787 | 0.7950 | 0.5842 | 0.0007 |
| aMAP score vs. THRI risk index | 0.3462 | 0.4502 | 0.3131 | 0.0374 | 0.5934 | 0.3564 |
| FIB-4 index vs. GES score | 0.3378 | 0.4066 | --- | 0.6479 | 0.8976 | 0.0005 |
| FIB-4 index vs. THRI risk index | 0.8705 | 0.9744 | --- | 0.5926 | 0.9427 | 0.3456 |
| GES score vs. THRI risk index | 0.2480 | 0.3620 | 0.2982 | 0.4701 | 0.9532 | 0.0288 |

**Supplementary table 4.** Harrel’s C index of respective risk stratification model by regions focusing on patients with cirrhosis at SVR

|  | Europe  (Italy, Germany, Austria, Switzerland) | North America  (USA) | South America  (Argentine, Brazil) | Middle East  (Egypt) | South and Southeast Asia  (India, Thailand)) | East Asia  (Japan, Taiwan) |
| --- | --- | --- | --- | --- | --- | --- |
| aMAP score | 0.602 | 0.502 | 0.658 | 0.762 | 0.578 | 0.516 |
| 95% C.I. | 0.568-0.636 | 0.456-0.548 | 0.598-0.718 | 0.748-0.776 | 0.554-0.602 | 0.500-0.532 |
| Number | 398 | 226 | 354 | 636 | 486 | 808 |
| HCC | 29 | 27 | 9 | 24 | 68 | 111 |
| FIB-4 index* | 0.592 | 0.522 | --- | 0.695 | 0.600 | 0.534 |
| 95% C.I. | 0.556-0.628 | 0.474-0.600 | --- | 0.672-0.718 | 0.582-0.618 | 0.520-0.548 |
| Number | 315 | 224 | --- | 654 | 543 | 813 |
| HCC | 25 | 27 | --- | 25 | 76 | 111 |
| GES score | 0.554 | 0.545 | 0.571 | 0.704 | 0.595 | 0.592 |
| 95% C.I. | 0.500-0.608 | 0.493-0.597 | 0.486-0.656 | 0.619-0.789 | 0.568-0.622 | 0.566-0.618 |
| Number | 346 | 210 | 251 | 254 | 516 | 822 |
| HCC | 30 | 26 | 8 | 11 | 75 | 119 |
| THRI risk index | 0.580 | 0.561 | 0.605 | 0.637 | 0.607 | 0.540 |
| 95% C.I. | 0.543-0.617 | 0.518-0.604 | 0.536-0.674 | 0.605-0.669 | 0.584-0.630 | 0.515-0.565 |
| Number | 427 | 226 | 387 | 655 | 542 | 813 |
| HCC | 29 | 27 | 11 | 25 | 76 | 111 |

*FIB-4 index could not be calculated in South American cohort due to unavailability of AST and ALT.

The model with highest C index of respective region was underlined.

**Supplementary table 5.** Harrel’s C index of respective risk stratification model by regions excluding patients with post-SVR HCC detected and diagnosed at advanced stage (BCLC 2–4)

|  | Europe  (Italy, Germany, Austria, Switzerland) | North America  (USA) | South America  (Argentine, Brazil) | Middle East  (Egypt) | South and Southeast Asia  (India, Thailand)) | East Asia  (Japan, Taiwan) |
| --- | --- | --- | --- | --- | --- | --- |
| aMAP score | 0.756 | 0.528 | 0.636 | 0.811 | 0.623 | 0.551 |
| 95% C.I. | 0.745-0.767 | 0.473-0.583 | 0.585-0.687 | 0.803-0.819 | 0.583-0.663 | 0.538-0.564 |
| Number | 597 | 272 | 1182 | 953 | 506 | 2715 |
| HCC | 4 | 19 | 18 | 19 | 17 | 136 |
| FIB-4 index* | 0.730 | 0.581 | --- | 0.728 | 0.666 | 0.545 |
| 095% C.I. | 0.717-0.743 | 0.528-0.634 | --- | 0.695-0.761 | 0.655-0.677 | 0.532-0.558 |
| Number | 406 | 279 | --- | 1011 | 560 | 2735 |
| HCC | 2 | 28 | --- | 21 | 18 | 137 |
| GES score | 0.540 | 0.551 | 0.680 | 0.708 | 0.710 | 0.641 |
| 95% C.I. | 0.365-0.715 | 0.494-0.608 | 0.593-0.767 | 0.601-0.815 | 0.669-0.751 | 0.618-0.664 |
| Number | 423 | 235 | 742 | 336 | 534 | 2775 |
| HCC | 3 | 18 | 9 | 7 | 18 | 142 |
| THRI risk index | 0.656 | 0.542 | 0.611 | 0.698 | 0.718 | 0.570 |
| 95% C.I. | 0.586-0.726 | 0.495-0.589 | 0.562-0.660 | 0.662-0.734 | 0.671-0.765 | 0.549-0.591 |
| Number | 659 | 272 | 1280 | 1012 | 560 | 2735 |
| HCC | 4 | 19 | 19 | 21 | 18 | 137 |

*FIB-4 index could not be calculated in South American cohort due to unavailability of AST and ALT.

The model with highest C index of respective region was underlined.

**Supplementary table 6.** Brier score of respective risk stratification model by regions

|  | Europe  (Italy, Germany, Austria, Switzerland) | North America  (USA) | South America  (Argentine, Brazil) | Middle East  (Egypt) | South and Southeast Asia  (India, Thailand)) | East Asia  (Japan, Taiwan) |
| --- | --- | --- | --- | --- | --- | --- |
| aMAP score | 0.0494 | 0.0245 | 0.0608 | 0.0431 | 0.0940 | 0.1027 |
| FIB-4 index* | 0.0472 | 0.1042 | --- | 0.1263 | 0.0928 | 0.1038 |
| GES score | 0.0518 | 0.1131 | 0.0170 | 0.1355 | 0.0860 | 0.1025 |
| THRI risk index | 0.0446 | 0.0213 | 0.0544 | 0.1163 | 0.0964 | 0.1029 |

*FIB-4 index could not be calculated in South American cohort due to unavailability of AST and ALT.

The model with lowest Brier score of respective region was underlined.
